# Evaluation of Leguminous Plants as Phytoremediator Species in Soil with Pesticide and Vinasse Interactions

**DOI:** 10.3390/plants14203137

**Published:** 2025-10-11

**Authors:** Munick Beato Aragão, Emanuella Roberto Ribeiro, Yanca Araujo Frias, Victor Hugo Cruz, Thalia Silva Valério, Alexandre Ribeiro Batista, Paulo Henrique Frata Ferreira, Henzo Henrique Simionatto, Paulo Renato Matos Lopes

**Affiliations:** 1 Department of Plant Production, College of Agricultural and Technological Sciences, São Paulo State University (UNESP), Dracena 17915-899, SP, Brazil; munick.aragao@unesp.br (M.B.A.); yanca.frias@unesp.br (Y.A.F.); hugo.cruz@unesp.br (V.H.C.); thalia.valerio@unesp.br (T.S.V.); ribeiro.batista@unesp.br (A.R.B.); frata.ferreira@unesp.br (P.H.F.F.); 2 Institute of Biosciences, Languages and Exact Sciences, São Paulo State University (UNESP), São José do Rio Preto 15054-000, SP, Brazil; emanuella.ribeiro@unesp.br; 3 Institute of Science and Technology, São Paulo State University (UNESP), Sorocaba 18087-100, SP, Brazil; henzo.h.simionatto@unesp.br

**Keywords:** bioremediation, *Canavalia ensiformis*, *Mucuna pruriens*, soil ecotoxicity, tebuthiuron, thiamethoxam

## Abstract

Sugarcane is a key crop for sugar, biofuels, and bioenergy, with Brazil as the world’s largest producer. Intensive cultivation demands pesticides like tebuthiuron and thiamethoxam, while fertigation with vinasse may alter their environmental behavior. Sustainable approaches, such as phytoremediation, are needed to mitigate negative impacts on soil quality. This study assessed the phytoremediation potential of *Canavalia ensiformis* and *Mucuna pruriens* in soils contaminated with tebuthiuron, thiamethoxam, and vinasse under greenhouse conditions. Experiments used a completely randomized design (five replicates, 4 × 2 factorial). Plant development impacts on the sentinel species *Crotalaria juncea*, and ecotoxicity via *Lactuca sativa* bioassays were evaluated. Tebuthiuron strongly inhibited *C. ensiformis*, while thiamethoxam showed mild stimulatory effects. *M. pruriens* maintained better growth in the presence of contaminants. Bioassays revealed greater residual toxicity in tebuthiuron treatments. Overall, *M. pruriens* demonstrated superior biomass production and capacity to lessen soil toxicity, underscoring its potential as a sustainable tool for phytoremediation of pesticide-impacted soils.

## 1. Introduction

Modern agriculture, focused on maximizing productivity, relies on the intensive and continuous use of chemical inputs to control factors that interfere with crop development, as well as on the reuse of agro-industrial by-products such as vinasse for fertigation [1]. In the cultivation of sugarcane (*Saccharum* spp.), a crop of major economic importance in Brazil and worldwide for the production of renewable biofuels, herbicides and insecticides are routinely applied to manage weeds and pests [2,3,4]. Among the pesticides widely used, tebuthiuron (1-(5-tert-butyl-1,3,4-thiadiazol-2-yl)-1,3-dimethylurea) stands out as a systemic herbicide of the phenylurea group that inhibits photosystem II, is absorbed by plant roots, and is translocated through the xylem [4]. Its high water solubility (2500 mg L^−1^) and low sorption coefficient (Kd = 1.32 and 0.85 L kg^−1^ for clayey and sandy soils, respectively) favor mobility in the soil profile, increasing the risk of leaching, aquifer contamination, and impacts on the soil microbiota [5]. This compound also shows high environmental persistence, with a degradation time of 90% (DT90) exceeding 330 days [4,6,7,8]. Another relevant pesticide is thiamethoxam (3-(2-chloro-1,3-thiazol-5-ylmethyl)-5-methyl-1,3,5-oxadiazinan-4-ylidene(nitro)amine), a systemic neonicotinoid insecticide applied for pest control and to promote plant vigor [9,10,11]. Its continuous use is concerning due to soil persistence of up to 618 days and high mobility, being classified as environmentally hazardous [10,11,12].

Beyond pesticide application, fertigation with vinasse is a common practice in the sugar-energy sector because this by-product is rich in organic matter and nutrients, contributing to soil fertility [13,14]. However, excessive use is associated with salinization, acidification, and phytotoxicity [15], as well as the risk of contaminating surface and groundwater through runoff or leaching [15,16]. Furthermore, the combined presence of pesticides and organic residues can lead to synergistic or antagonistic interactions, the so-called “cocktail effect”. Ref. [17] showed that pesticide mixtures enhance structural and functional changes in lipid membranes, and [18] reported that the interaction among tebuthiuron, thiamethoxam, and vinasse significantly alters soil microbial activity, including antagonism of thiamethoxam when applied with vinasse.

Sugarcane is typically cultivated under continuous cropping systems, where the same area is replanted for successive production cycles without long fallow periods. This management involves repeated pesticide applications and vinasse fertigation, resulting in persistent chemical loads and changes in soil microbial communities over time. Although the present study did not use soil collected directly from sugarcane fields, the experimental design simulated the chemical pressures of continuous cropping by applying field-recommended rates of tebuthiuron, thiamethoxam, and vinasse while controlling for pre-existing residues. This strategy allows for an isolated evaluation of plant–contaminant interactions under conditions relevant to sugarcane production.

Given the persistence of these compounds and the difficulty of predicting their combined effects, it is essential to adopt strategies that restore soil functionality and reduce environmental risks. Phytoremediation, which uses plants with high biomass production and vigor to extract, degrade, or immobilize pollutants, stands out as an economically viable and low-impact technology capable of interacting positively with rhizospheric microorganisms [19]. The efficiency of this technique depends on the selection of species that tolerate and attenuate contaminants through bioaccumulation, degradation, or stabilization [20]. Among these options, legumes such as *Mucuna pruriens* and *Canavalia ensiformis*, traditionally used as green manure, have documented potential for soils contaminated by pesticides, while also providing agronomic benefits such as increased organic matter and nitrogen incorporation, which favor soil quality recovery and crop rotation [7,21,22].

In this context, the present study aimed to evaluate the tolerance and phytoremediation capacity of *M. pruriens* and *C. ensiformis* in soils containing tebuthiuron and thiamethoxam, with or without vinasse, through an integrated analysis, contributing to sustainable strategies for the recovery of agricultural areas with residual pesticide concentrations.

## 2. Results

### 2.1. Growth and Biomass of Leguminous Species

**Figure 1:**
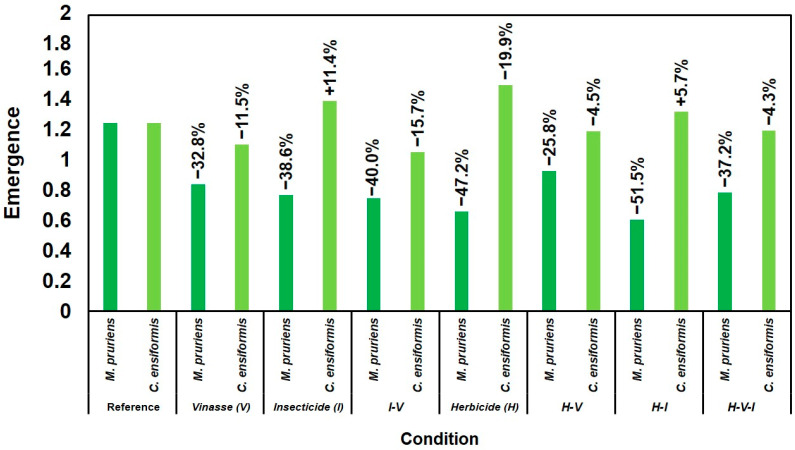
Analysis of seedling emergence for *C. ensiformis* and *M. pruriens* in different soil samples.

The emergence of *Mucuna pruriens* (MP) was significantly lower than that of the control, indicating its sensitivity to the compounds present in the soil (Figure 1). The herbicide–insecticide (H–I) combination reduced MP emergence by 51.45%, highlighting the combined negative effect of the compounds on the species’ initial development. In contrast, *Canavalia ensiformis* (CE) showed a higher emergence rate under all conditions, particularly in treatments containing only H, where there was a 20% increase compared to the control.

**Figure 2:**
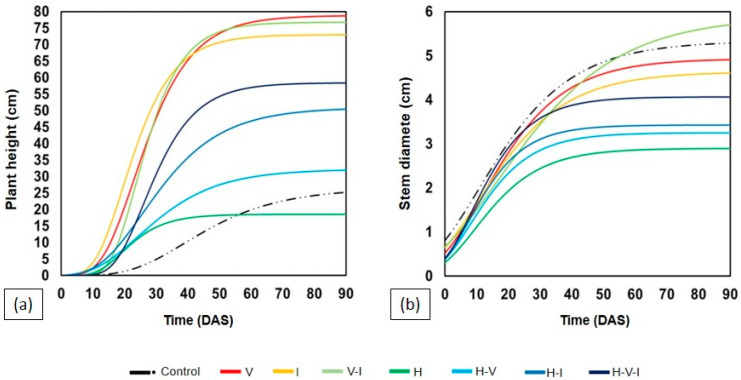
Monitoring of *M. pruriens* development with growth projected up to 90 DAS using the Gompertz model in the different soil samples: (**a**) plant height, and (**b**) stem diameter. Tebuthiuron (H), Thiamethoxam (I), and Vinasse (V).

MP development was monitored over 70 days after sowing (DAS), with projections up to 90 DAS using the Gompertz model (Figure 2). The presence of H in the soil resulted in a clear phytotoxic effect during the first weeks, significantly reducing plant height until approximately 35 DAS. After this period, partial growth recovery was observed in treatments where H was combined with I or with the combination of I and V (H–V–I), surpassing the performance of the treatment with H alone. Statistical analyses at 70 DAS confirmed that soils with H alone showed significantly lower development than those without H (control, V, I, and V–I), as well as the combination with I (H–I). A similar pattern was observed for stem diameter, in which the absence of H resulted in more uniform values among treatments, while the presence of H caused marked reductions (Table 1).

**Figure 3:**
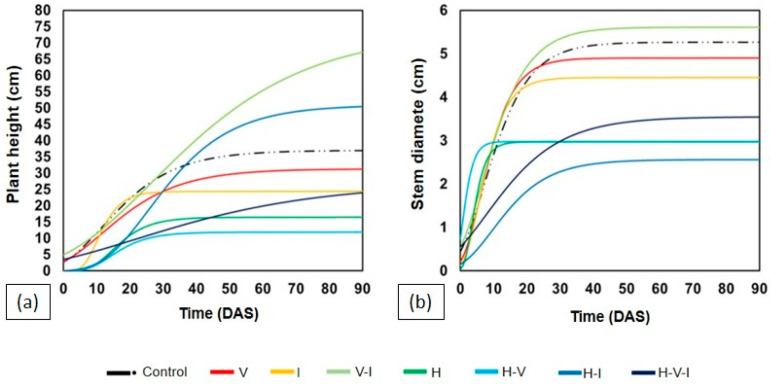
Monitoring of plant development of *C. ensiformis* with estimation at 90 DAS using the Gompertz model in different soil samples: (**a**) plant height, and (**b**) stem diameter. Tebuthiuron (H), Thiamethoxam (I), and Vinasse (V).

The development of CE was strongly affected by the presence of H (Figure 3). Most treatments containing this compound resulted in early plant death, except for the treatment combining H, I, and V, in which the plants were able to survive until the end of the cycle. In treatments without herbicide, such as those with V and/or I (V, I, V–I), CE showed continuous and vigorous growth, with height and stem diameter comparable to the control. The combination of V and I showed the best performance among the non-herbicide treatments (Table 2).

In treatments with H alone or in combination with I and V, plant death was observed by 12 DAS, preventing statistical analysis (Table 2).

**Figure 4:**
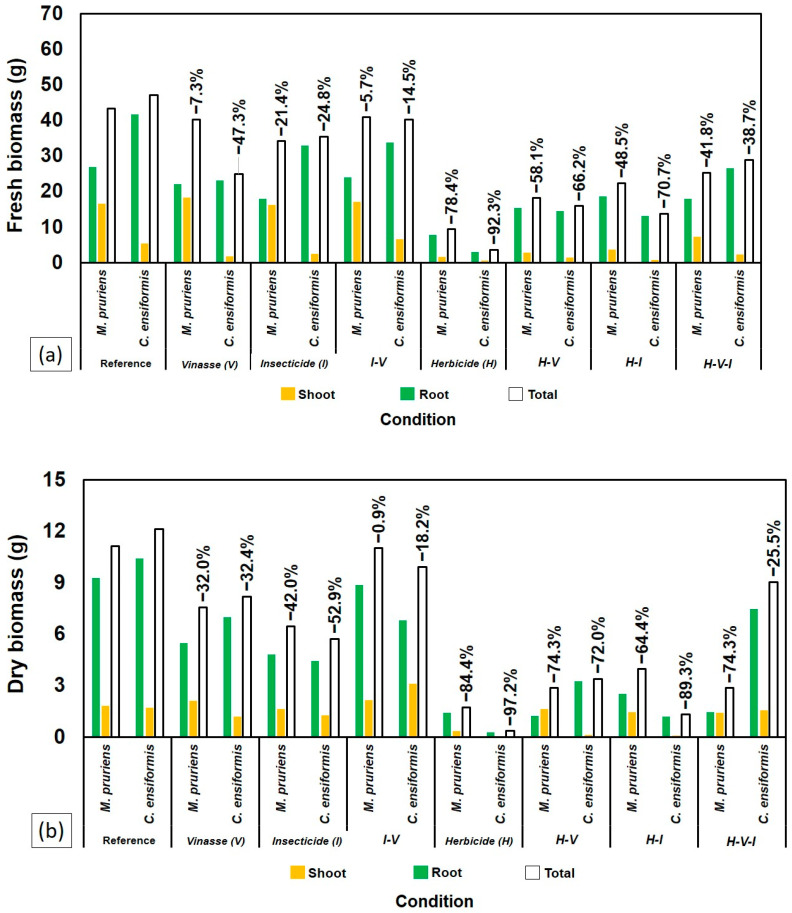
Comparison between *C. ensiformis* and *M. pruriens* after 70 DAS in different soil samples regarding biomass: (**a**) fresh and (**b**) dry.

At 70 DAS, the plants were harvested to assess the shoot and root fresh and dry biomass. Both species showed sensitivity to the presence of H, with significant reductions in fresh biomass (Figure 4a) and dry biomass (Figure 4b) compared to the control and other treatments without H. The treatment with H alone resulted in the lowest biomass values, highlighting the high phytotoxicity of the compound for both legumes.

Despite the general sensitivity to H, CE exhibited higher dry biomass production when all three compounds (H-V-I) were combined, outperforming MP in this specific context (Figure 4b).

### 2.2. Sentinel Species Response

**Figure 5:**
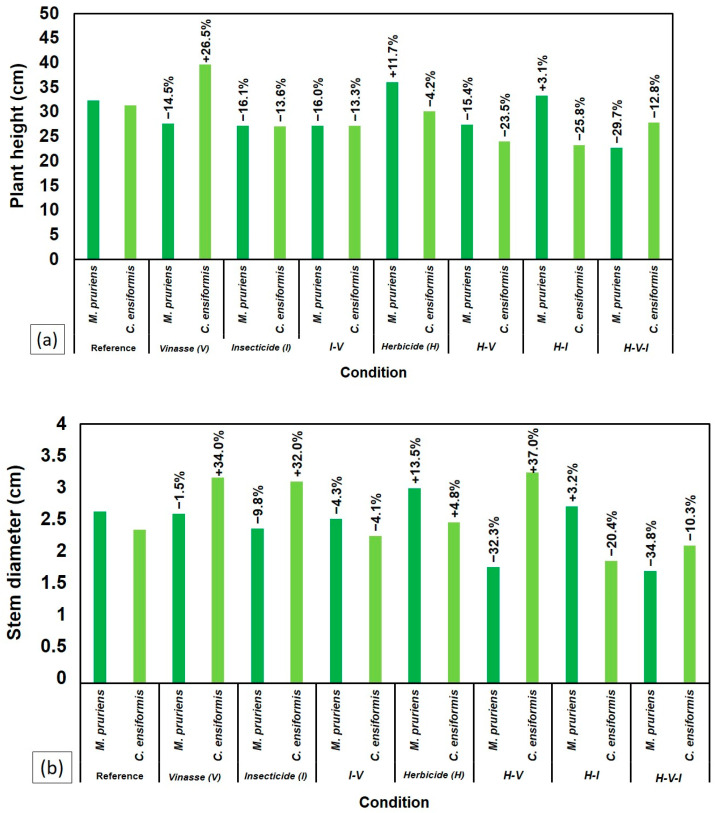
Plant development of *Crotolaria juncea* at 61 DAS in different soil samples previously cultivated with *C. ensiformis* and *M. pruriens*: (**a**) plant height, and (**b**) stem diameter.

After the cultivation of the remediating species, the development of *Crotalaria juncea* (CJ) was monitored for 61 days as an indicator of residual soil toxicity. CJ height was higher in the samples that had received V and prior cultivation of CE, surpassing soils with MP by 46.38% and the control by 26.45% (Figure 5a).

**Figure 6:**
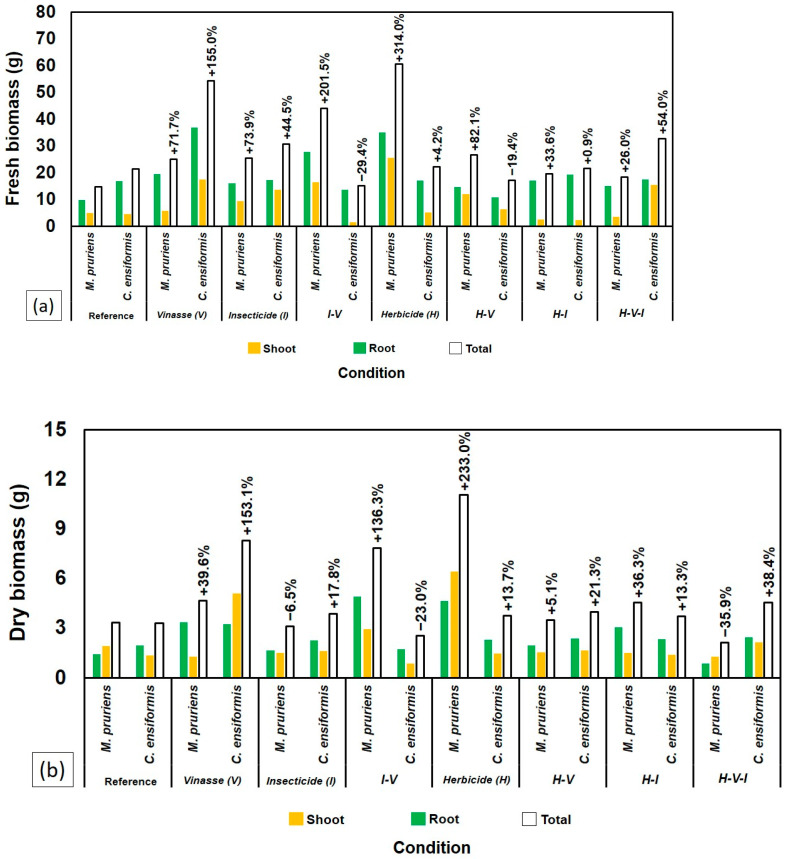
Shoot fresh (**a**) and dry (**b**) biomass of *Crotalaria juncea* at 61 DAS in different soil samples previously cultivated with *C. ensiformis* and *M. pruriens*.

However, biomass behavior showed the opposite trend: the highest accumulations of shoot fresh and dry biomass occurred in soils previously cultivated with MP, especially in treatments with H alone (260.7%) and with I combined with V (185.4%) compared to the reference (Figure 6a). Similar results were observed for shoot dry biomass (Figure 6b).

### 2.3. Ecotoxicological Bioassays and Multivariate Analyses

**Figure 7:**
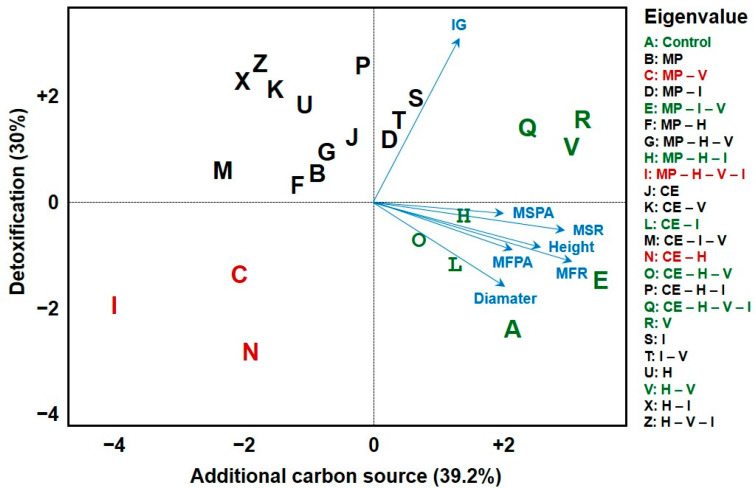
Principal component analysis of *Crotalaria juncea* morphological data and ecotoxicological bioassays in different soil samples previously cultivated with *C. ensiformis* (CE) and *M. pruriens* (MP). H = tebuthiuron; I = thiamethoxam; V = vinasse; MP = *Mucuna pruriens*; CE = *Canavalia ensiformis*.

To complement plant growth results, ecotoxicological bioassays with Lactuca sativa were conducted after the cultivation of MP and CE. Principal component analysis (PCA) (Figure 7) revealed correlations between morphological and ecotoxicological variables, grouping the treatments with the best CJ performance (control, MP–I–V, MP–H–I, CE–I, and CE–H–V). These groups showed reduced residual toxicity and better plant development, suggesting effective contaminant attenuation by the legumes.

**Figure 8:**
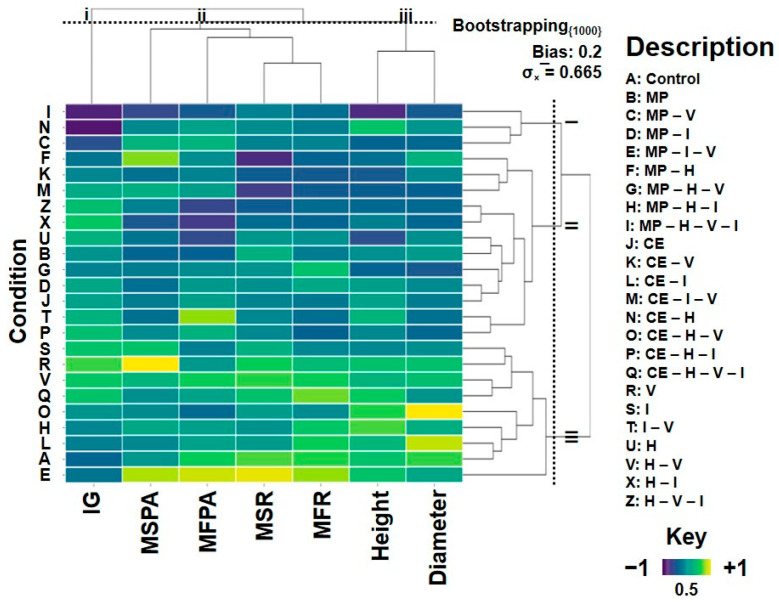
Heatmap of *Crotalaria juncea* morphological data and germination index from ecotoxicity bioassays in different soil samples previously cultivated with *C. ensiformis* (CE) and *M. pruriens* (MP). H = tebuthiuron; I = thiamethoxam; V = vinasse; MP = *Mucuna pruriens*; CE = *Canavalia ensiformis*; CJ = *Crotalaria juncea*.

Conversely, treatments with MP–V, MP–H–V–I, and CE–H exhibited low L. sativa germination indices and reduced CJ growth, indicating higher residual toxicity. The heatmap (Figure 8) confirmed these trends, highlighting poor phytoremediation performance in treatments with excessive vinasse or high herbicide content.

Despite the sensitivity of CE to H (which caused death under several conditions), some CE treatments displayed reduced soil toxicity in the bioassays. Treatments with CE–H–I–V and CE–H–I, for example, showed lower toxicity to L. sativa than the corresponding MP treatments, indicating that CE may promote beneficial soil interactions under certain contaminant combinations.

## 3. Discussion

### 3.1. Phytotoxic Effects and Species Tolerance

The results shown in Figure 1 and Figure 2 and Table 1 clearly demonstrate distinct tolerance levels between the two leguminous species studied. *Mucuna pruriens* (MP) showed higher ability to maintain growth and biomass in soils containing tebuthiuron (H) and thiamethoxam (I), whereas *Canavalia ensiformis* (CE) exhibited pronounced sensitivity, with early plant death under most herbicide-containing treatments (Figure 3 and Table 2).

The strong phytotoxic effect of tebuthiuron on CE corroborates the findings of [22], who also reported complete plant death under similar conditions up to 29 days after sowing. Tebuthiuron’s inhibitory action on photosystem II and its persistence in soil [5,6] explain these patterns.

Conversely, MP showed partial growth recovery after initial inhibition by H, particularly when combined with I and vinasse (V), as illustrated in Figure 2. This resilience likely arises from physiological plasticity and MP’s capacity to maintain rhizospheric microbial activity, which can accelerate herbicide degradation [7,22]. The superior biomass accumulation of MP under stress conditions (Figure 4) reinforces its role as a suitable phytoremediator. These findings align with [8] and [23], who demonstrated that MP cultivation reduces residual tebuthiuron toxicity, especially when integrated with microbial bioaugmentation.

The initially lower emergence rate of MP compared with CE (Figure 1) may reflect its natural dormancy and slower germination [24]. Nevertheless, once established, MP sustained continuous growth and produced greater biomass, highlighting adaptive mechanisms that favor persistence in contaminated soils.

### 3.2. Influence of Vinasse and Compound Interactions

Vinasse (V) exerted a marked influence on both plant development and ecotoxicological responses. Moderate vinasse doses generally improved plant growth and alleviated herbicide toxicity, while complex or excessive combinations with pesticides led to increased residual toxicity.

As seen in Figure 2 and Figure 4, the combination of H and V impaired plant growth less severely than H alone, suggesting a partial buffering effect. This observation agrees with [7], who found that vinasse, when applied at suitable volumes (≈150 m^3^ ha^−1^), can mitigate tebuthiuron phytotoxicity by serving as a nutrient source.

However, under the combined presence of all contaminants (H–V–I), MP showed reduced root development (Figure 6), and *Crotalaria juncea* (CJ) bioassays revealed residual toxicity. These findings align with the “cocktail effect” proposed by [17], in which pesticide mixtures cause synergistic or antagonistic outcomes that modify toxicity.

Ref. [18] also demonstrated that vinasse alters the environmental behavior of tebuthiuron and thiamethoxam—sometimes inhibiting microbial respiration with I, but enhancing mineralization when applied with H. This mechanism likely explains the intermediate performance seen in Figure 3 and Figure 6, where certain combinations (H–V–I) improved plant tolerance and reduced toxicity despite the presence of herbicides.

These complex outcomes highlight that the combined application of agro-industrial residues and pesticides cannot be linearly predicted: their environmental impact depends on compound interactions, soil conditions, and microbial adaptation.

### 3.3. Microbial and Environmental Implications of Phytoremediation

The superior performance of MP in treatments combining H, I, and V (Figure 4, Figure 5 and Figure 6) underscores its potential for use in phytoremediation of pesticide-contaminated soils. Its persistence under contaminant stress suggests an efficient association with rhizospheric microorganisms capable of transforming or immobilizing pollutants.

Continuous sugarcane cropping systems, simulated in this experiment, are known to alter soil microbial composition [25,26]. Thus, the observed resilience of MP may reflect enhanced microbial–plant cooperation under such chemical pressures. The PCA analysis (Figure 7) and heatmap (Figure 8) further support this, showing clustering of MP treatments with reduced soil toxicity and improved *Crotalaria juncea* performance.

These ecotoxicological findings confirm that MP can effectively reduce residual toxicity, in agreement with [22,23,27], who reported similar improvements in germination index and plant development when using legumes as remediating species.

Nevertheless, treatments containing high vinasse and herbicide concentrations still exhibited elevated toxicity (Figure 8), suggesting that vinasse’s organic overload can interfere with nutrient uptake or microbial balance, as also described by [7]. According to [28], such organic residues can alter adsorption and degradation kinetics, sometimes hindering pesticide dissipation.

Interestingly, even though CE suffered high mortality under H (Figure 3), treatments like CE–H–I–V displayed lower toxicity in *Lactuca sativa* assays (Figure 8). This implies that CE roots, even when stressed or decaying, may stimulate microbial activity that contributes to detoxification. Therefore, combining tolerant (MP) and sensitive (CE) legumes might offer a synergistic phytoremediation strategy, enhancing overall soil recovery through microbial and biochemical diversity.

## 4. Materials and Methods

### 4.1. Location and Materials

The experiment was performed under greenhouse conditions from September to December 2023 at 21°28′57″ S and 51°31′58″ W—Dracena, Brazil.

To simulate the chemical pressures of continuous sugarcane cropping, the doses of tebuthiuron, thiamethoxam, and vinasse were defined based on field agronomic recommendations, applied as a single treatment but in proportions that reproduce the chemical conditions found in sugarcane fields subjected to successive production cycles. This approach allowed the evaluation of plant–contaminant interactions in a controlled manner, without the interference of pre-existing residues.

The soil Utisol [29] collected up to 0.20 m in Dracena (Brazil) was from a degraded pasture area with no recent history of pesticide application. The analytical characteristics were: phosphorus (1 mg dm^−3^), organic matter (4 g dm^−3^), potassium (0.3 mmolc dm^−3^), calcium (2 mmolc dm^−3^), magnesium (6 mmolc dm^−3^), aluminum (13 mmolc dm^−3^), potential acidity [H+Al] (33 mmolc dm^−3^), sum of bases (8 mmolc dm^−3^), cation exchange capacity (41 mmolc dm^−3^), bases saturation (20%), aluminum Saturation (61%), sulfur (7 mg dm^−3^), and pH (4.0). For treatments without vinasse, soil amendment was performed using lime (PRNT 95%) (1800 kg ha^−1^), single superphosphate (222 kg ha^−1^), and potassium chloride (25 kg ha^−1^).

*Mucuna pruriens* (MP) and *Canavalia ensiformis* (CE) were selected for their use in sugarcane crop succession and their ability to remediate pesticide-contaminated soil [22], while *Crotalaria juncea* (CJ) was used as a sentinel species [30]. All seeds were purchased from Sementes Piraí^®^ (Piracicaba, São Paulo, Brazil).

Vinasse was obtained from a sugarcane energy plant at Dracena, Brazil, applied manually and uniformly, simulating the fertigation technique, according to CETESB P4.231 [31] guidelines, considering the soil potassium content. A volume of 400 mL per pot was used, corresponding proportionally to the recommended field dose.

Pesticides were applied according to the manufacturers’ recommendations and soil characteristics. Tebuthiuron (Combine^®^, Zionsville, India, 500SC, 500 g a.i. L^−1^, Dow AgroSciences^®^, Zionsville, India) and thiamethoxam (Actara^®^ 750SG, 700 g a.i. L^−1^, Syngenta^®^) were purchased from a commercial establishment in Dracena (São Paulo, Brazil) and used at rates of 1.0 L ha^−1^ and 1000 g ha^−1^, respectively. Pesticides application was via an automatic laboratorial device, which provided a suite of Magno ADGA-03 flat nozzles every 0.5 m over a 1.0 m bar for broadcast spraying. A configurated sprayer released pesticides at 200 kPa, 0.65 L min^−1^, 5 km·h^−1^ and 0.75 m high from the top of the pots.

### 4.2. Experimental Design and Analytical Methods

The experiment was conducted using a randomized block design, with five replicates for each treatment, based on the presence or absence of three compounds: tebuthiuron (herbicide, H), thiamethoxam (insecticide, I), and vinasse (V). Three plant species conditions were evaluated (absence, *M. pruriens*—MP, and *C. ensiformis*—CE), totaling 120 experimental units.

Experimental units consisted of 4.0 L pots filled with air-dried and sieved soil after fertility correction. Following pesticide application according to the experimental design, vinasse was applied manually and uniformly to the surface, simulating fertigation.

Seven days later, three seeds were sown per pot. On the 12th day after sowing (DAS), thinning was performed, leaving one plant per pot. Irrigation was carried out daily according to water demand. Plants were grown until 70 DAS, when they were harvested. Subsequently, the sentinel species *C. juncea* (CJ) was sown in the same pots and cultivated for an additional 61 DAS under the same conditions.

During cultivation, weekly measurements of plant height and stem diameter were taken. At the end of the cycle, shoor and root fresh and dry biomass were determined.

To assess the residual toxicity of the treatments, ecotoxicological bioassays were performed using *Lactuca sativa* seeds. Soil from the pots was collected at the end of the growth period of the potential phytoremediator species (70 DAS), and an aqueous extract was prepared according to NBR 10.006 [32]. The procedure followed the methodology described by [33] based on seed germination and seedling development, allowing the calculation of the germination index (GI). Such ecotoxicological bioassays are essential to complement the evaluation of remediation efficiency, as they reveal residual biological effects in the soil, as evidenced in previous studies from our group [7,22,27].

### 4.3. Statistical Analysis

Data were evaluated for distribution and variance homogeneity using normality tests and Bartlett’s homoscedasticity tests, followed by analysis of variance with Tukey’s test (*p* ≤ 0.05). For modeling growth over time, the Gompertz model was applied [34], which allows the description of vegetative growth patterns in time series, as used by [18,22,27].

Statistical analyses were performed using R software (version 4.0.2; R Core Team, Vienna, Austria, 2020) [35], including the generation of a heatmap in which color scales represented the relative intensity of morphological and ecotoxicological parameters evaluated for each treatment. This approach enabled an integrated visualization of the effects caused by the presence of contaminants and plant species [36].

## 5. Conclusions

*Mucuna pruriens* not only tolerates and attenuates tebuthiuron and thiamethoxam more effectively than *Canavalia ensiformis,* but may also contribute to the restructuring of soil conditions and consequently favor the establishment of subsequent crops. Its superior vegetative development, along with a potential ability to influence the soil microbial community, suggests that *M. pruriens* could reduce the risk of phytotoxicity in continuous cropping systems and enhance the sustainability of agricultural management.

Soil samples containing thiamethoxam, either alone or in combination with vinasse, supported plant growth and helped mitigate the toxicity impact. Despite the benefit of its presence in some combinations, vinasse increased residual toxicity when associated with the pesticides. Thus, ecotoxicity bioassays confirmed these patterns, which showed lower potential damage in soils previously cultivated with *M. pruriens*, particularly when combining thiamethoxam and vinasse.

Overall, the findings highlight *M. pruriens* as a promising species for the phytoremediation of soils contaminated with tebuthiuron and thiamethoxam, especially when vinasse is applied in balanced amounts. They also emphasize the need for further research on pesticide–organic matter interactions and on the possible effects of *M. pruriens* on microbial community reorganization, which may directly influence remediation processes.

## Figures and Tables

**Table 1:** Analysis of variance of the morphological data of *M. pruriens* after 70 DAS in the different soil samples: plant height, stem diameter, fresh biomass, and dry biomass.

Treatments	*Mucuna pruriens*							
	Plant Height (cm)		Stem Diameter (mm)		Fresh Biomass (g)		Dry Biomass (g)	
**Control**	70.75	a	5.63	a	38.75	a	10.88	a
**V**	67.60	a	4.90	ab	38.23	a	7.34	abc
**I**	67.00	a	4.73	ab	31.13	a	6.44	abc
**V–I**	76.25	a	5.46	ab	43.73	a	9.68	ab
**H**	23.75	b	4.49	ab	7.77	b	1.75	c
**H–V**	40.50	ab	3.83	b	7.45	b	5.53	abc
**H–I**	68.33	a	4.39	ab	22.86	ab	5.02	abc
**H–V–I**	63.50	ab	5.46	ab	23.97	ab	3.70	bc

Different lowercase letters indicate significant differences among treatment means within the column (Tukey test at 5.0% significance level). Tebuthiuron (H), Thiamethoxam (I), and Vinasse (V).

**Table 2:** Analysis of variance of the morphological data of *C. ensiformis* after 70 DAS in the different soil samples: plant height, stem diameter, fresh biomass, and dry biomass.

Treatments	*Canavalia ensiformis*							
	Plant Height (cm)		Stem Diameter (mm)		Fresh Biomass (g)		Dry Biomass (g)	
**Control**	43.00		5.61	ab	46.08	a	11.68	ab
**V**	27.33		6.14	ab	11.54	b	2.66	b
**I**	39.00		5.34	b	32.89	ab	8.76	ab
**V–I**	41.00		7.59	a	50.37	a	14.75	a
**H**	ND		ND		ND		ND	
**H–V**	ND		ND		ND		ND	
**H–I**	ND		ND		ND		ND	
**H–V–I**	27.50		5.80	ab	35.55	ab	7.03	ab

Different lowercase letters indicate significant differences among treatment means within the column (Tukey test at 5.0% significance level). ND—Not detected due to plant death. Tebuthiuron (H), Thiamethoxam (I), and Vinasse (V).

## Data Availability

The authors declare that AI was used only to improve the quality of the writing and that the authors assume responsibility for the accuracy and originality of the text.
